# Five Cases of Hepatic Mesenchymal Tumors Diagnosed by Endoscopic Ultrasound‐Guided Tissue Acquisition

**DOI:** 10.1002/deo2.70268

**Published:** 2025-12-28

**Authors:** Yuichi Takano, Naoki Tamai, Jun Noda, Tetsushi Azami, Fumitaka Niiya, Tatsuya Yamagami, Akihiro Nakamura, Genshu Tate, Takafumi Ogawa, Masatsugu Nagahama

**Affiliations:** ^1^ Department of Internal Medicine Division of Gastroenterology Showa Medical University Fujigaoka Hospital Yokohama Japan; ^2^ Department of Diagnostic Pathology Showa Medical University Fujigaoka Hospital Yokohama Japan

**Keywords:** endo‐hepatology, endoscopic ultrasound, liver, mesenchymal tumor, tissue acquisition

## Abstract

Percutaneous biopsy remains the gold standard for diagnosing focal liver lesions; however, endoscopic ultrasound‐guided tissue acquisition (EUS‐TA) has recently emerged as a promising alternative. Although its diagnostic performance is favorable, most available evidence has focused on epithelial tumors, and reports on mesenchymal tumors are rare. Herein, we report five cases of hepatic mesenchymal tumors diagnosed using EUS‐TA. The cohort comprised four male and one female patient, with a median age of 70 years (range, 63–88). The median targeted lesion size was 30 mm (range, 22–61 mm), predominantly located in the left lateral hepatic segments. Four cases underwent transgastric biopsy and one transduodenal biopsy, using either a 22‐gauge aspiration or biopsy needle. All procedures were technically successful, and no procedure‐related adverse events occurred. Histopathological examination established definitive diagnoses of leiomyosarcoma (*n* = 2), angiosarcoma (*n* = 1), epithelioid hemangioendothelioma (*n* = 1), and metastatic gastrointestinal stromal tumor (*n* = 1). Subsequent management included chemotherapy in three patients and best supportive care in two. This case series demonstrates that EUS‐TA is a feasible diagnostic modality for hepatic mesenchymal tumors, providing sufficient tissue for histological and immunohistochemical evaluation.

## 1 Introduction

Percutaneous liver biopsy (PLB) has traditionally been the first‐line method for obtaining histological diagnoses of focal liver lesions. In recent years, the concept of *Endo‐hepatology* has gained widespread acceptance, with endoscopic ultrasound‐guided tissue acquisition (EUS‐TA) emerging as a promising alternative diagnostic modality [1]. A recent meta‐analysis reported that the diagnostic yield of EUS‐TA for focal liver lesions was as high as 92.4%, with adverse event rates ranging from 0% to 6% [2]. Nonetheless, most previous studies have primarily focused on epithelial tumors, including metastatic liver tumors, hepatocellular carcinoma, and intrahepatic cholangiocarcinoma, while reports on mesenchymal tumors remain scarce. In general, the histopathological diagnosis of mesenchymal tumors is more complex than that of epithelial tumors, often requiring immunohistochemical (IHC) analysis in addition to hematoxylin and eosin (H&E) staining. It remains unclear whether hepatic mesenchymal tumors can be reliably diagnosed using specimens obtained via EUS‐TA. Here, we report five cases of hepatic mesenchymal tumors that were successfully diagnosed using EUS‐TA.

## 2 Case Presentation

**FIGURE 1:**
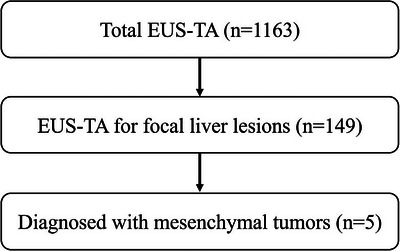
Patient flowchart showing the number of endoscopic ultrasound‐guided tissue acquisition (EUS‐TA) procedures performed between 2016 and 2024, the subset performed for focal liver lesions, and the number ultimately diagnosed as mesenchymal tumors.

Between January 2016 and December 2024, five cases of hepatic mesenchymal tumors diagnosed using EUS‐TA were retrospectively reviewed at our institution (Figure 1). Among the five patients, four were male, and one was female, with a median age of 70 years (range: 63–88). The median diameter of targeted lesions was 30 mm (range: 22–61 mm). Four tumors were located in the left lateral segment of the liver (Segments II and III), and one in the medial segment (Segment IV) (Table 1).

**TABLE 1:** Clinical characteristics of five cases of hepatic mesenchymal tumors diagnosed by endoscopic ultrasound‐guided tissue acquisition (EUS‐TA).

Case	Age	Sex	Final diagnosis	Location	Diameter (mm)	Puncture site	Needle gauge	Needle type	Cytological diagnosis	Treatment	Clinical outcomes
1	#	M	Leiomyosarcoma (Primary)	SegmentII	30	Transgastric	22	Expect	Class III	Best supportive care	dead (9 months)
2	#	M	Leiomyosarcoma (Metastatic)	SegmentII	61	Transgastric	22	Top Gain	Class II	Chemotherapy (Adriamycin)	dead (3 months)
3	#	F	Angiosarcoma (Primary)	SegmentII	24	Transgastric	22	Expect	Class II	Chemotherapy (at another hospital)	N/A
4	#	M	Epithelioid hemangioendothelioma (Primary)	Segment IV	51	Transduodenal	22	Top Gain	Class II	Best supportive care	dead (4 months)
5	#	M	GIST (Metastatic)	SegmentII	22	Transgastric	22	Top Gain	Class I	Chemotherapy (Imatinib)	alive (31months)
											N/A: not available

EUS‐TA was performed under conscious sedation using pethidine hydrochloride (35 mg) and midazolam (1.5–3.5 mg). A GF‐UCT260 echoendoscope (Olympus Medical Systems, Tokyo, Japan) and either a UE‐ME1 or UE‐ME2 ultrasound processor (Olympus Medical Systems) were used.

In four of the five cases, tissue acquisition was performed via the transgastric approach, whereas in one case involving a lesion in Segment IV, the transduodenal approach was employed. Two patients underwent biopsy with a 22‐gauge Expect SlimLine needle (Boston Scientific Japan, Tokyo, Japan), and three with a 22‐gauge SonoTip TopGain needle (Medico's Hirata, Tokyo, Japan). The number of needle strokes ranged from 10 to 20, and a negative suction pressure of 10–20 mL was applied. Rapid on‐site cytological evaluation was not performed. No procedure‐related adverse events, such as bleeding, perforation, or infection, were observed (Supporting information).

**FIGURE 2:**
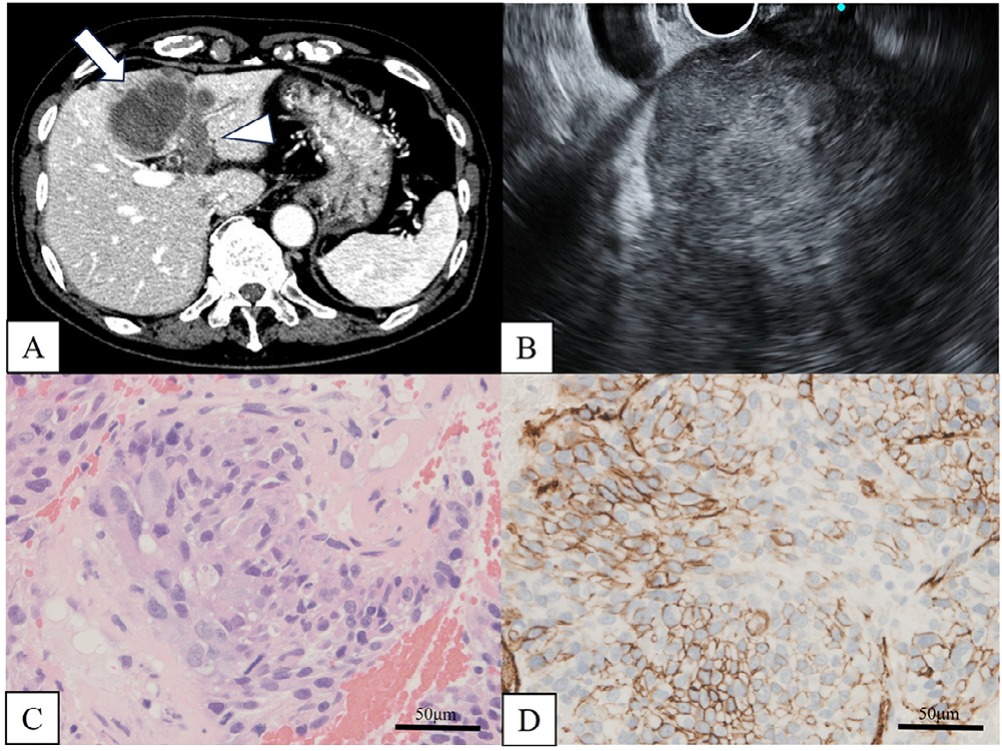
(A) An 88‐year‐old man (Case 4) underwent contrast‐enhanced computed tomography (CT), which revealed a 51‐mm hypovascular tumor in hepatic segment IV (arrow) with tumor thrombus in the left portal vein (arrowhead). (B) Endoscopic ultrasound (EUS) from the duodenal bulb demonstrated a heterogeneous mass with mixed hyperechoic and hypoechoic areas. EUS‐guided tissue acquisition (EUS‐TA) was performed using a 22‐gauge needle. (C) Spindle‐shaped to polygonal tumor cells were arranged in an epithelioid growth pattern. The tumor cells had abundant eosinophilic cytoplasm with evident mitotic figures. Some tumor cells contained cytoplasmic vacuoles, resembling primitive vascular lumina (H&E stain, ×400). (D) The tumor cells were positive for CD34 (CD34 immunostaining, ×400). Taken together, these histomorphological and immunohistochemical findings led to the diagnosis of hepatic epithelioid hemangioendothelioma.

Tissues obtained from EUS‐TA were fixed in formalin, followed by histological diagnosis using H&E staining. After the tissue was fixed in formalin, the remaining liquid component was cytologically examined with Papanicolaou staining. All tissue specimens obtained by EUS‐TA were considered adequate for cytological and histological analysis. Cytological evaluation showed benign findings in four cases (Class I in one case, Class II in three), and an intermediate result (Class III) in one case. No cytological evidence of malignancy was observed. Histopathological diagnosis revealed two cases of leiomyosarcoma (one primary hepatic, one metastatic from a retroperitoneal lesion), one case of primary hepatic angiosarcoma, one case of primary hepatic epithelioid hemangioendothelioma, and one case of metastatic gastrointestinal stromal tumor (GIST) originating from the small intestine. IHC staining was performed in all cases (Table 2 and Figure 2).

**TABLE 2:** Details of immunohistochemical staining.

		Epithelial marker			Muscle marker		Vascular marker		Neural marker	GIST marker		Proliferation index
Case	Final Diagnosis	CKAE1/3	CK7	CK20	SMA	h‐caldesmon	CD31	CD34	S‐100	C‐kit	DOG1	Ki‐67
1	Leiomyosarcoma	—	N/A	N/A	+	+	N/A	—	—	—	—	40%
2	Leiomyosarcoma	—	N/A	N/A	+	N/A	N/A	—	—	—	—	30%–40%
3	Angiosarcoma	+	—	—	—	N/A	+	—	—	—	—	N/A
4	Epithelioid hemangioendothelioma	—	±	—	N/A	N/A	+	+	N/A	N/A	—	N/A
5	GIST	N/A	N/A	N/A	+	N/A	N/A	+	—	+	+	5%–10%
												N/A: not available

## 3 Discussion

In recent years, several studies have demonstrated the usefulness of EUS‐TA as an alternative to PLB. Yane et al. [3] reported comparable diagnostic accuracy between EUS‐TA and PLB (100% vs 90%), but noted postprocedural pain (7.1%) and one case of needle tract seeding (2.4%) in the PLB group, whereas EUS‐TA was associated with one bleeding event (3.6%). Another comparative study found similar diagnostic accuracy (100% vs 96%) but significantly fewer adverse events with EUS‐TA (2% vs 17%) [4], with most PLB‐related events being pain and one case of bleeding requiring interventional radiology. Although data remain limited, EUS‐TA provides diagnostic performance comparable to PLB with potentially fewer adverse events.

EUS‐TA provides access to anatomically challenging regions such as the caudate lobe and is performed under sedation without skin incision, resulting in reduced patient discomfort and less postprocedural pain compared with PLB [3, 4, 5]. Although the left hepatic lobe and caudate lobe are most commonly targeted through a transgastric approach, right lobe lesions can also be accessed transduodenally [6]. Furthermore, because EUS provides access from within the gastrointestinal tract, it is less affected by ascites present on the liver surface, and the use of a thinner needle compared with PLB may contribute to a lower risk of bleeding. However, EUS‐TA cannot be performed in patients with pharyngeal or esophageal strictures, surgically altered anatomy (e.g., total gastrectomy), or severe respiratory failure preventing sedation. It also requires procedural expertise and is limited to facilities equipped with EUS.

The decision to perform EUS‐TA rather than PLB was based on a combination of anatomical and institutional factors. From an anatomical standpoint, all lesions were located in the left hepatic lobe and were readily accessible using EUS. At our institution, EUS‐TA is actively employed for focal liver lesions, and it is particularly regarded as the first‐line diagnostic approach for lesions located in the left hepatic lobe and caudate lobe. There were no cases in which PLB was attempted unsuccessfully prior to performing EUS‐TA in this study.

According to the World Health Organization (WHO) Classification of Tumors, 5th Edition: Digestive System Tumors [7], mesenchymal tumors represent a heterogeneous group of neoplasms. They include GISTs, lipomatous and fibroblastic tumors, smooth and skeletal muscle tumors, vascular tumors, and rare entities of uncertain differentiation, such as perivascular epithelioid cell tumors and hamartoma. The clinical utility of EUS‐TA has been firmly established for GISTs, which are frequently encountered in routine practice [8].

In this study, five hepatic mesenchymal tumors were successfully diagnosed using EUS‐TA with IHC, indicating its diagnostic utility for these rare lesions. Advances in biopsy needles, including Franseen, reverse‐bevel, and fork‐tip designs, have improved tissue core acquisition. Zhao et al. demonstrated that fine‐needle biopsy (FNB) outperforms fine‐needle aspiration (FNA) in IHC adequacy (82.4% vs. 66.7%) and diagnostic accuracy (74.4% vs. 55.4%) [9]. In our series, FNB was used in three cases, likely contributing to diagnostic success, supporting the proactive use of FNB when mesenchymal tumors are suspected.

This study spans a 9‐year period (2016–2024). At our institution, FNA needles were used until 2021, after which FNB needles became standard; therefore, needle selection largely reflected this chronological shift. In collaboration with our pathologists, we compared FNA and FNB specimens and found no difference in specimen adequacy or IHC feasibility. However, given the small sample size, further evaluation in a larger cohort is warranted. Considering both prior evidence [9] and our institutional experience, the FNB needle is recommended for obtaining sufficient tissue for IHC, and we particularly favor the TopGain needle due to its technical advantage in achieving a steeper puncture angle.

Adequate specimens were obtained in all cases, likely due to increased use of the FNB needle, and the submission of all solid material for histopathological analysis. When sample volume was limited, essential IHC stains were prioritized, and if tissue remained insufficient, additional sampling was performed using an FNB needle; in selected cases, PLB was also considered. While a clear minimum tissue threshold could not be defined, our pathologists indicated that a ≥5‐mm core generally enables adequate histopathological and IHC evaluation, even for rare mesenchymal tumors.

Although all five lesions were ultimately malignant mesenchymal tumors, cytology was negative in every case. This reflects our practice of using solid tissue for histology and performing cytology only on the remaining fluid, resulting in low cellularity. Additionally, mesenchymal tumors typically exhibit minimal cytologic atypia and limited exfoliation, inherently reducing cytologic sensitivity. These observations underscore the necessity of obtaining sufficient core material for histopathological and IHC assessment rather than relying on cytology.

In conclusion, we report five cases of hepatic mesenchymal tumors successfully diagnosed by EUS‐TA. Because these tumors are rare and lack reliable serum markers or pathognomonic imaging features, histological diagnosis remains essential. Our findings suggest that EUS‐TA may represent a valuable diagnostic modality even for these uncommon hepatic tumors.

## Author Contributions

**Conceptualization and resources**: Yuichi Takano; **writing – original draft**: Yuichi Takano; **writing – review & editing**: all authors.

## Funding

The author has received no specific funding for this work.

## Consent

The patients provided informed consent for the publication of this case report.

## Conflicts of Interest

The authors declare no conflicts of interest.

## Supporting information




**Supporting File 1**: deo270268‐sup‐0001‐SuppMat.pdf
